# Advancing the inclusion of pregnant and lactating populations in HIV PrEP research: ethical, regulatory, and surveillance recommendations from a multisectoral working group

**DOI:** 10.3389/fpubh.2026.1811039

**Published:** 2026-07-06

**Authors:** C. E. Osakwe, R. Schaefer, L. Donaldson, A. S. Abiodun, M. Chatani-Gada, A. K. Chigome, S. Day, C. Deaton, U. G. Elemuwa, H. Ingold, D. Joseph Davey, K. Kersey, S. Lamprianou, A. D. Lyerly, L. M. Noguchi, P. P. M. Nyambayo, C. Mpaso, M. N. Robertson, F. Renaud, T. Sehloho, J. van Wyk, V. Vannappagari, M. Warren, V. Miller, on behalf of the Long-Acting PrEP during Pregnancy and Lactation Working Group

**Affiliations:** 1 Forum for Collaborative Research, University of California, Berkeley, DC, United States; 2 National Agency for Food and Drug Administration and Control (NAFDAC), Abuja, Nigeria; 3 Independent Global Health Consultant, Longmeadow, MA, United States; 4 South African Health Products Regulatory Authority, Pretoria, South Africa; 5 Department of Medicine, Division of Infectious Diseases, the University of North Carolina at Chapel Hill, Chapel Hill, NC, United States; 6 Gilead Sciences, Inc., Foster, CA, United States; 7 Global HIV, Tuberculosis, Viral Hepatitis, and STIs Programmes, World Health Organization, Geneva, Switzerland; 8 Division of Infectious Diseases, School of Medicine, University of California Los Angeles, Los Angeles, CA, United States; 9 Department of Epidemiology and Biostatistics, School of Public Health, University of Cape Town, Cape Town, South Africa; 10 Pharmacovigilance Team, Department of Regulation and Prequalification, World Health Organization, Geneva, Switzerland,; 11 Department of Social Medicine and Center for Bioethics, University of North Carolina at Chapel Hill, Chapel Hill, NC, United States; 12 Jhpiego, Johns Hopkins University, Washington, DC, United States; 13 Medicines Control Authority of Zimbabwe, Harare, Zimbabwe; 14 Pangaea Zimbabwe, Harare, Zimbabwe; 15 BDI Consulting, LLC., Nipomo, CA, United States; 16 ViiV Healthcare, Durham, NC, United States; 17 AVAC, New York, NY, United States

**Keywords:** breastfeeding, HIV, pre-exposure prophylaxis, pregnancy, women

## Abstract

Despite a 60% decline in new HIV infections since 1995, significant challenges persist. In 2023, 1.3 million new HIV infections occurred, with 645,500 in sub-Saharan Africa, where cisgender adolescent girls and women represent over two-thirds of new cases. Approximately one-quarter of vertical HIV transmissions are linked to infections during pregnancy and lactation, yet data on PrEP use in these populations remains generally limited due to their exclusion from clinical trials, despite a recent movement toward inclusion, such as in the PURPOSE 1 trials evaluating the efficacy and safety of lenacapavir. This data gap is concerning given that women face an elevated risk of HIV acquisition during pregnancy. Despite this heightened risk, access to PrEP remains restricted among these groups. This article, based on insights from a multi-regional and multi-sectoral working group convened by the Forum for Collaborative Research between January 2024 and February 2025, addresses this critical issue. The working group, comprising stakeholders from regulatory agencies, research, industry, and communities, held meetings and a scientific symposium to inform evidence-based strategies for improving the inclusion of pregnant and lactating women in PrEP trials. The working group identified key barriers, including ethical concerns, limited safety data, minimal community engagement, regulatory gaps, weak incentives, and limited surveillance capacity. They recommend reframing pregnant and lactating women as needing protection through research, alongside standardized data systems, stronger incentives, global collaboration, and digital innovation to ensure equitable inclusion in HIV prevention efforts.

## Introduction

Global HIV prevention has advanced with antiretroviral drugs used as pre-exposure prophylaxis (PrEP), which markedly reduce HIV risk when taken as prescribed. Yet despite a 60% decline in new infections since 1995, 1.3 million new cases were reported in 2023 (1). Over half of these, 645,500, were in sub-Saharan Africa, with cisgender adolescent girls and women representing over two-thirds of those new infections (1). Despite a significant scale-up of PrEP globally, only 3.5 million people used PrEP at least once in 2023, which is well below the UNAIDS target of 21.2 million PrEP users by 2025 (1).

HIV acquisition risk for women is particularly high during pregnancy and the postpartum period (2). Approximately one-quarter of vertical HIV transmissions globally is attributed to HIV infections that occur during pregnancy and lactation (3). To curb HIV and eliminate vertical transmission, prevention must prioritize women of childbearing potential and ensure access during pregnancy and lactation (4), particularly because there are considerable gaps in access to and use of PrEP during pregnancy and lactation (5). In this article, we examine the historical exclusion of pregnant and lactating women from clinical trials, recent advances in inclusive HIV prevention research, and the need for post-marketing surveillance, drawing on discussions from a multi-regional, multi-sectoral working group convened by the Forum for Collaborative Research (The Forum). The Forum, a public–private partnership at the University of California, Berkeley, was tasked with convening US, European, and African regulators and stakeholders to advance pre- and post-approval data collection on PrEP use in pregnant and lactating women. To facilitate dialog and exchange, a working group was formed, consisting of representatives from regulatory agencies, international organizations, academia, industry, and community and advocacy groups This article highlights the challenges and recommendations identified by the working group. Table 1 lists the organizations and expertise represented by the authors; all working group members and their affiliations are listed under Acknowledgments.

**Table 1:** Affiliation and expertise of authors writing on behalf of the working group.

Organization	Representative	Expertise
WHO Global HIV, TB, Viral Hepatitis and STI Programmes	H Ingold	HIV Prevention Programmes and Triple Elimination of HIV, HBV and Syphilis
WHO Global HIV, TB, Viral Hepatitis and STI Programmes	F Renaud	Toxicity Monitoring and Person-Centered Care
WHO Division of Pharmacovigilance	S Lamprianou	Pharmacovigilance
Medical Control Agency Zimbabwe (MCAZ)	PPM Nyambayo	Pharmacovigilance and Mobile Health Adverse Event Surveillance
National Agency for Food and Drug Administration and Control (NAFDAC)	AS Abiodun	Pharmacovigilance Specialist
	UG Elemuwa	Director of Pharmacovigilance
South African Health Products Regulatory Authority	AK Chigome	Medicine Regulatory Officer - Pharmacovigilance
	T Sehloho	Senior Clinical Evaluation Manager
Pangaea, Zimbabwe	C Mpaso	Reproductive Health and HIV Prevention
AVAC	M Warren	Community Engagement, Advocacy, Research Literacy and GPP
Gilead Sciences	C Deaton	Patient Safety
	K Kersey	Clinical Development
ViiV	V Vannappagari	Epidemiology and Real World Data
	J van Wyk	Regulatory and Safety Governance
University of Cape Town	D Joseph Davey	Epidemiology and Biostatistics
University of California, Los Angeles	D Joseph Davey	Infectious Disease Medicine and Epidemiology
University North Carolina Chapel Hill	S Day	Sociology and Stakeholder Engaged Research
	AD Lyerly	Social Medicine and Bioethics
Jhpiego	LM Noguchi	Maternal and Newborn Safety in HIV Prevention
Consultants	M Chatani-Gada	Global Health-Research Literacy
Forum for Collaborative Research	E Osakwe	Public Health
	R Schaefer	Epidemiology and International Policy
	L Donaldson	Public Health
	MN Robertson	Clinical Development, Infectious Diseases
	V Miller	Regulatory Science

## The evolution of women participation in HIV research

Historically, women, especially if pregnant or lactating, were excluded from clinical trials for new drugs due to concerns about reproductive health impacts and ethical complexities (6). The Helsinki Declaration, established in 1964, is a set of ethical guidelines for the involvement of human participants in medical research (7). This Declaration labeled pregnant and lactating women as vulnerable, limiting their research participation, and in 1977 the US FDA similarly recommended excluding women of childbearing potential from early drug trials (8). By excluding women from clinical research, safety and efficacy data on pharmaceutical products were commonly not made available for them (9). In response to the women’s movement, the 1980s Women’s Health Task Force, and evidence that biological and demographic factors affect drug response, the US FDA’s 1988 guideline required safety and efficacy analyses by age, sex, and race (10). This led to a 1993 FDA guideline on evaluating sex differences in drug trials, calling for women’s inclusion in clinical research (11). The 1993 US Revitalization Act mandated inclusion of women and minorities in federally funded research, yet pregnant and lactating women remain underrepresented, leaving major evidence gaps in HIV (12–14).

In 1992, out of 14,799 participants enrolled in the AIDS Clinical Trials Group studies, only 1,151 were women (6). A systematic review of HIV trials (1994–2011) found median female participation of 19.2% in ARV studies, 38.1% in vaccine trials, and 11.1% in cure research (15). This lack of representation in HIV clinical research is even more significant for pregnant and lactating women. An analysis of the International Clinical Trials Registry Platform from January 2001 to December 2015 showed that, on average, HIV therapies were studied during pregnancy and lactation 4.4 years after the first regulatory approval (16). Delays in pharmacokinetic data were longer, estimated at 6 years, for drugs studied in pregnancy (17). Limited pregnancy and lactation data at approval often lead to cautious labeling, restricting access to new HIV products or prompting use without adequate evidence.

## Advancements in the inclusion of pregnant and lactating women in HIV research

More recently, initiatives such as the PHASES Project (“Ending the Evidence Gap for Pregnant Women Around HIV and Co-Infections”) have called for a key conceptual shift from protecting pregnant and lactating women *from* research to protecting them *through* research (18). In 2020–2022, WHO and the International Maternal Pediatric Adolescent AIDS Clinical Trials Network (IMPAACT) convened a series of technical workshops that included researchers, funders, regulators, clinical experts, industry leaders, civil society, ethicists (19). A new framework for accelerating the study of new antiretrovirals during pregnancy was proposed and key principles were disseminated through a Call to Action (13, 20). This framework suggests that if an HIV prevention drug is effective in non-pregnant individuals and drug exposure levels are similar during pregnancy, its prevention efficacy can be applied to pregnant populations, provided existing safety data supports it.

Oral tenofovir disoproxil fumarate/emtricitabine (TDF/FTC) and long-acting injectable cabotegravir (CAB-LA), have shown remarkable efficacy in preventing HIV acquisition, with efficacy studies completed in non-pregnant populations. Oral TDF/FTC is the most studied and has shown good safety and tolerability for mothers and infants (21, 22). Large CAB-LA efficacy trials excluded pregnant women and required contraception, limiting pregnancy-specific data; however, in the open-label extension of HPTN 084, participants who became pregnant could continue injections, with findings suggesting CAB-LA was safe and well tolerated, and maternal and infant outcomes comparable to the general population (23). While pregnant and lactating women were initially excluded from the placebo-controlled clinical trials on the dapivirine vaginal ring (DVR), safety and pharmacokinetic studies have since been conducted for pregnant and lactating populations. All three studies conducted among pregnant women, lactating women, and breastfeeding mother-infant pairs have demonstrated that the DVR is safe for these populations (24–26). Twice-yearly injections with lenacapavir (LEN) demonstrated 100% efficacy against HIV acquisition in the PURPOSE 1 study among cisgender adolescent girls and young women (16 to 24 years old) in Uganda and South Africa (27, 28). The PURPOSE trial collected pregnancy safety and pharmacokinetic data by not requiring contraception and allowing participants who became pregnant to continue the study drug after reconsent (27). At the time of an interim analysis, there were 193 pregnancies among those randomized to LEN and available pregnancy outcomes were similar to those expected for the population (27).

## The need for PrEP surveillance during pregnancy and lactation

Although clinical research evaluates the safety and efficacy of pharmaceutical products, they are carried out under controlled conditions that do not necessarily provide data on product use under real-world conditions (29). Clinical trial participants are usually carefully selected, meeting specific requirements, which may limit the generalizability of the results (30). Studies often have limited duration and sample sizes, hindering the detection of long-term or rare drug effects. This necessitates post-marketing surveillance to monitor real-world PrEP use in pregnant and lactating individuals. Such surveillance is crucial for identifying less common and long-term pregnancy, maternal, and infant health outcomes, which can then inform clinical decisions and improve guidelines. Despite this critical need, post-approval research and surveillance of PrEP use during pregnancy and lactation are generally lacking (31). Research is particularly lacking on long-term developmental outcomes in infants exposed to PrEP in utero or via breastfeeding, with few studies assessing effects beyond 1 year post-exposure (16).

## Working group processes and identified challenges and recommendations

The Forum invited representatives from regulatory and other governmental agencies, international organizations, industry, research, and community and advocacy groups at the end of 2023. The working group met nine times between January 2024 and February 2025. In addition, the working group co-organized a scientific symposium at the 5th HIV Research for Prevention Conference in Lima, Peru, 6–10 October 2024. Apart from the public symposium, working group meetings were by invitation only to ensure frank and open dialog. The Forum followed a standard process in facilitating the working group, as described elsewhere (32). A writing group drafted this article the organizational relevance and expertise is described in Table 1, and all members of the working group are listed under Acknowledgments. Participants were invited to join the working group based on their area of expertise and their ability to represent the key stakeholder groups that the Forum works with, including community advocates, regulatory authorities, clinician scientists and academic researchers, international organizations, and industry. All participants engaged openly and were identified to other members of the group. There was no formal consensus (e.g., Delphi) nor evidence grading applied. All opinions expressed during discussions were considered and are reflected in the recommendations presented in this article. Disagreements or divergent views were resolved through continued dialog, and where full agreement was not reached, the range of perspectives has been captured in the recommendations. Table 2 summarizes the challenges and recommendations identified by the working group. A writing group composed of a representational subset of working group members drafted this article and was reviewed by most working group members.

**Table 2:** Challenges, progress made, and actionable strategies regarding generating data on pregnant and lactating women in pre-exposure prophylaxis (PrEP) clinical trials and surveillance.

Challenges	Recommended strategies for action
Inclusion of pregnant and lactating women in clinical trials	
Ethical complexities and paradigm shifts	Reconceptualizing pregnant and lactating women as a clinically complex (rather than vulnerable) population, emphasizing the need to protect this population through equitable participation in HIV research.
Limited and timely availability of pre-clinical//clinical safety evidence	Collecting pregnancy- and lactation-specific data throughout the drug development process, including reproductive toxicology studies in animal models, pharmacokinetic and pharmacodynamic studies.
Minimal community involvement in clinical trial design	Engaging with civil societies, community advocates, and local communities affected by HIV across all clinical trial phases will ensure that the trial protocols align with community needs, concerns, and priorities.
Lack of regulatory clarity and harmonization	Clarifying regulatory pathways to ensure product labeling with clear messaging on safety of PrEP use during pregnancy and lactation, including updating labels after product approval; harmonization of regulatory requirements can facilitate standardized clinical research data collection (e.g., under the ICH E21) (62).
Few incentives for conducting research involving pregnant and lactating women	Creating incentives and establishing requirements for research by regulatory agencies can encourage sponsors to conduct pregnancy- and lactation-related research and mobilize investment in this area.
Limited technical capacity for regulatory review	Strengthening capacity building initiatives, knowledge exchange, harmonization of regulatory standards across countries and regions, the use of regulatory reliance, and the use of digital collaboration tools for secure data sharing and joint evaluations.
Surveillance of PrEP use during pregnancy and lactation	
Lack of standardized data collection processes and harmonized systems in post-approval studies	Standardizing protocols and centralizing data collection systems using innovative digital tools for post-approval studies and surveillance (e.g., the WHO Pregnancy Therapeutic Working Group has recently produced standardized outcomes and shares materials from more than 23 studies to harmonize and accelerate research and surveillance in pregnancy in the ARV in pregnancy toolkit) (14).
Limited surveillance capacity	Strengthening surveillance systems through collaborations that support knowledge exchange, regulatory harmonization and reliance, and the use of digital tools.
Minimal community involvement in post-approval surveillance	Increasing community engagement and partnership with civil society organizations to ensure timely and accurate reporting of adverse outcomes.

## Challenges and recommendations for including pregnant and lactating women in PrEP clinical trials and post-approval surveillance

### Ethical complexities and paradigm shifts

A key challenge in including pregnant and lactating women in PrEP trials is addressing ethical concerns about maternal and infant safety; the working group highlighted PHASES’ efforts to develop ethical guidance for HIV research in pregnancy (18). The guidance reframed pregnant women as complex rather than vulnerable, emphasizing protection through research and equitable inclusion over presumptive exclusion (18). Advocacy by pregnant and lactating women shaped this guidance, with the working group endorsing the PHASES project’s call to build research capacity, promote innovative trial designs for inclusion, and prioritize pregnancy-specific pharmacokinetic studies in drug development and post-approval safety monitoring (18). This guidance aligns with other initiatives, including the WHO/IMPAACT call to action (13).

Despite shifts toward protection through research, informed consent gaps remain, especially in LMICs where limited health literacy may impede understanding of PrEP risks and benefits (33). Additionally, in some settings, male partners or family members influence women’s health decisions, affecting their autonomy (34). Addressing these gaps requires simplified, locally translated consent processes and community engagement strategies that empower women to make informed choices.

### Limited availability of pre-clinical/clinical safety evidence

A major barrier to including pregnant and lactating women in HIV trials is the limited and delayed availability of data on reproductive safety, teratogenicity, and pharmacokinetics, which often results in their early exclusion and the loss of critical pregnancy- and lactation- specific evidence (35). Prioritizing pre-clinical data by conducting reproductive toxicology studies in animal models helps in generating preliminary safety data specific to pregnancy and lactation (36). Following preclinical animal studies, pharmacokinetic and pharmacodynamic research is needed to guide dosing in pregnant and lactating women (37).

When pregnancy- and lactation-specific data are collected during pre-clinical and early clinical stages of development, clinical safety data on the use of PrEP in pregnant and lactating women can be generated through the inclusion of these populations in pivotal registration trials, as exemplified by the LEN phase 3 trial among cisgender women that allowed participants who became pregnant to continue receiving study drugs. Innovative designs such as adaptive trials with dose adjustments and combined phase I/II studies may be needed to generate pregnancy- and lactation-specific pharmacokinetic, safety, and efficacy data (38). Outside HIV prevention research, the IMPAACT 2001 combined phase I/II trial evaluated the pharmacokinetics, tolerability, and safety of rifapentine and isoniazid for latent tuberculosis in pregnant women with and without HIV (39). The study showed that 12 once-weekly doses of rifapentine and isoniazid can be used in late pregnancy without dose adjustment, despite differences in drug clearance (39). Preliminary data suggest rifapentine and isoniazid are safe in pregnancy, but the working group cautioned that early dose adjustments based on limited data should be made carefully to avoid adverse effects in later trials or post-approval use.

### Minimal community involvement in clinical trial design

Limited community engagement in trial design risks excluding input from pregnant and lactating women, resulting in protocols misaligned with their needs. The Good Participatory Practice guidelines (GPP) developed by UNAIDS and AVAC in 2007 and updated in 2011 to include HIV biomedical prevention provide systematic guidance on engaging relevant stakeholders in the design and conduct of HIV prevention clinical trials (31). Participatory approaches improve enrollment and persistence in PrEP trials (40). Engaging directly with the communities in all stages of clinical trials, from study design to results dissemination, ensures that research is respectful, relevant, and aligns with the needs of affected populations (41, 42). The PURPOSE 1 study protocol was deliberate about the inclusion of pregnant women, allowing participants to make informed decisions regarding the use of the study drug during pregnancy (43). Stakeholder priorities were determined and addressed through stakeholder consultations in South Africa and Uganda, where the study was conducted (43), and a Global Community Accountability Group was instrumental in guiding the development of the study protocol. This process provides a benchmark for future HIV clinical trials.

### Lack of regulatory clarity and harmonization

Trial sponsors and the pharmaceutical industry face significant challenges in navigating varied regulatory requirements for PrEP drug labels that permit use during pregnancy and lactation. This leads to poor standardization of data collection in clinical trials and a lack of harmonized guidelines across regulatory agencies, contributing to a reluctance to include pregnant and lactating women in research. To rectify this, clearer and harmonized regulatory guidance is needed, along with sensitization campaigns for regulatory agencies to reduce restrictions on pregnant populations in research. The African Medicines Agency (AMA), an African Union specialized agency, aims to enhance access to quality, safe, and effective medical products across Africa. It can foster harmonization on the continent by directly reviewing medical products and coordinating joint reviews under its regional authority (44). Clear regulatory definitions are needed to ensure accurate PrEP labeling for pregnancy and lactation, enabling ethical research that minimizes harm and maximizes benefit (45). Marketing authorization holders of PrEP products are also urged to file with regulatory authorities in a timely matter for updated product labels as new data becomes available.

### Few incentives for conducting research on pregnant and lactating women

Including pregnant and lactating women in trials requires added resources due to regulatory and logistical complexities and retaining them through pregnancy and postpartum follow-up is challenging (38). Providing incentives is key to motivating clinical trial sponsors to include pregnant and lactating women in research. Regulatory bodies, like the US FDA, could encourage this through policies similar to the Best Pharmaceuticals for Children Act (BPCA), which offers patent extensions, and the Pediatric Research Equity Act (PREA), which mandates data collection for pediatric populations (45). Incentives and clearer regulatory requirements for pregnancy- and lactation-specific data can drive investment and motivate sponsors to generate evidence in these populations (46).

### Limited technical capacity for regulatory reviews

Regulators face challenges reviewing new PrEP products for pregnancy and lactation due to limited safety data, compounded by resource and expertise constraints in many national agencies (46). Addressing these challenges will require strengthening capacity, for instance through global partnerships and regional knowledge sharing, collaborative and innovative approaches such as the harmonization of regulatory standards across countries and regions and the use of regulatory reliance (such as those processes proposed under the AMA), and the use of digital collaboration tools for secure data sharing and joint evaluations (46–49). Global partnerships, such as the EU-Medicine for All (EU-M4All) program and WHO prequalification and collaborative registration procedures, can accelerate product registration, while multistakeholder collaboration among industry, regulators, WHO, academia, and civil society is essential to strengthen capacity through technical exchange.

### Lack of standardized data collection processes and harmonized systems in post-approval studies and systems

Post-approval surveillance systems vary widely across countries and are commonly limited due to insufficient resources. Existing surveillance systems are usually fragmented, which prevents the pooling of data from different sources (45). PrEP surveillance is undermined by non-standardized data and unclear indicators for pregnant and lactating women, while poor interoperability across research platforms, Electronic Health Records (EHRs), and national health systems limits longitudinal tracking and real-world evidence generation (50).

The working group emphasized that standardized and harmonized data collection and systems in post-approval research and surveillance are required to ensure quality data and facilitate data synthesis (19). Tailored digital health platforms specifically for pregnant and lactating populations can help capture relevant pharmacovigilance, adherence, and safety data in real time (51). The WHO research toolkit offers standardized pharmacovigilance guidance for maternal and infant outcomes, promoting data quality, harmonization, and coordinated safety signal evaluation (14). Standardized data protocols and centralized repositories are needed to systematically collect safety data on PrEP use in pregnant and lactating women (52). Strengthening pharmacovigilance and reporting requires leveraging global collaborative systems that monitor and collect adverse drug reactions. Key examples include VigiBase®, the US FDA Adverse Event Reporting System (FAERS), the Task Force on Research Specific to Pregnant and Lactating Women (PRGLAC), the WHO Advisory Committee on Safety of Medicinal Products (ASCoMP), and EudraVigilance, Europe’s medicine safety database (53–57). Post-marketing surveillance could further be harmonized using a network approach, as is done for HIV prevention clinical trials.

### Limited surveillance capacity

Many regulatory agencies lack the capacity and secure digital infrastructure needed for comprehensive surveillance, including for products used in pregnancy and lactation. Strengthening PrEP surveillance requires collaboration, knowledge exchange, harmonization, and reliance mechanisms. The AMA seeks to advance regulatory harmonization, including for surveillance in Africa (49). The AMA is also poised to strengthen surveillance within the region by coordinating information exchange among member countries (49). The African Union’s Smart Safety Surveillance (AU-3S) program monitors priority medicines and supports the AMA by strengthening pharmacovigilance through innovation, capacity building, resource pooling, and collaboration with national regulators (58). The use of digital tools, such as mobile applications for reporting adverse drug reactions and electronic health records for data integration and storage, enhances real-time data collection, analysis, and communication for effective safety monitoring (59). Artificial Intelligence tools can enhance pharmacovigilance by improving signal detection and predicting adverse reactions, enabling proactive safety surveillance (60, 61). The working group also noted the importance of integrating PrEP surveillance into broader surveillance systems as opposed to siloed approaches.

### Minimal community engagement in post-approval surveillance

The working group emphasized stronger community and civil society engagement in surveillance of PrEP effectiveness and safety post-approval. This is needed to build trust and ensure culturally appropriate data collection methods. Actively engaging with and strengthening the capacity of communities will also contribute to accurate and timely reporting of adverse events or longer-term risks of PrEP use during pregnancy and lactation. This is imperative for the success of transitioning from regarding pregnant women as “vulnerable – needing protection from research” to “informed because of participation in research.”

## Conclusion

Including pregnant and lactating women in PrEP research is essential to close data gaps strengthen regulation, surveillance, community engagement for HIV prevention and ultimately reducing HIV infections.

Advancing PrEP access in pregnancy and lactation requires stronger collaboration across regulators, governments, researchers, providers, communities, and industry, alongside increased funding for implementation research and surveillance, greater use of real-world data, and a human right–based approach to trial design and policy.

Ensuring pregnant and lactating women are meaningfully involved in research and decision-making is key to sustainable, equitable HIV prevention. This working group demonstrates how multistakeholder collaboration can bridge gaps, build consensus, and advance equitable PrEP access for these populations.

## Data Availability

The raw data supporting the conclusions of this article will be made available by the authors, without undue reservation.

## Group members of the Long-acting PrEP during pregnancy and lactation working group

Abiola A., National Agency for Food and Drug Administration and Control, Abuja, Nigeria; Badshah C., Merck, Rahway, NJ; Belew Y., U. S. Food and Drug Administration, Silver Spring, MD; Chan H., Merck, Rahway, NJ; Chatani M., Independent Global Health Consultant; Chi B., University of North Carolina at Chapel Hill, Chapel Hill, NC; Chigome A., South African Health Products Regulatory Authority, Pretoria, South Africa; Davey D., University of California, Los Angeles, CA; Day S., University of North Carolina at Chapel Hill, Chapel Hill, NC; Deaton C., Gilead Sciences, Inc., Foster City, CA; Delaney-Moretlwe S., University of the Witwatersrand, Johannesburg, South Africa; Donaldson L., Forum for Collaborative Research, Washington, DC; Elemuwa U., National Agency for Food and Drug Administration and Control, Abuja, Nigeria; Escudeiro Dos Santos M., European Medicines Agency, Amsterdam, Netherlands; Force L., Gilead Sciences, Inc., Foster City, CA; Gonzalez Tome M., European Medicines Agency, Madrid, Spain; Hamm Rush S., Gates Foundation, Seattle, WA; Ikhide Y., National Agency for Food and Drug Administration and Control, Abuja, Nigeria; Ingold H., World Health Organization, Geneva, Switzerland; John-Stewart G., University of Washington, Seattle, WA; KERSEY K., Gilead Sciences, Inc., Foster City, CA; Kumwenda G., AVAC, Southern Region, Malawi; Lamprianou S., World Health Organization, Geneva, Switzerland; Lataillade M., Gates Foundation, Hartford, CT; Lievense B., AVAC, New York, NY; Love J., Merck, Rahway, NJ; Lyerly A., University of North Carolina at Chapel Hill, Chapel Hill, NC; Makura C., Pangaea Zimbabwe, Harare, Zimbabwe; Matthews L., University of Alabama at Birmingham, Birmingham, AL; Mgodi N., University of Zimbabwe, Harare, Zimbabwe; Miller V., Forum for Collaborative Research, Washington, DC; Moodley D., University of KwaZulu-Natal, Durban, South Africa; Morroni C., University of Edinburgh, Edinburgh, Scotland; Mujugira A., Makerere University, Kampala, Uganda; Mullick C., U. S. Food and Drug Administration, Silver Spring, MD; Ndagije H., National Drug Authority, Kampala, Uganda; Ndembi N., International Vaccine Institute, Kigali, Rwanda; Noguchi L., Johns Hopkins University, Baltimore, MD; Nwokike J., United States Pharmacopeia, North Bethesda, MD; Nyambayo P., Medicines Control Authority of Zimbabwe, Harare, Zimbabwe; Osakwe C. E., Forum for Collaborative Research, Washington, DC; Plank R., Merck, Rahway, NJ; Prochazkz Nunez M., World Health Organization, Geneva, Switzerland; Ranade R., Forum for Collaborative Research, Washington, DC; Renaud F., World Health Organization, Geneva, Switzerland; Rinehart A., ViiV Healthcare, Washington, DC; Robertson M., BDI Consulting, Nipomo, CA; Rodolph M., World Health Organization, Geneva, Switzerland; Saidi F., University of North Carolina, Lilongwe, Malawi; Schaefer R., Forum for Collaborative Research, Washington, DC; Sehloho T., South African Health Products Regulatory Authority, Pretoria, South Africa; Semete B., South African Health Products Regulatory Authority, Pretoria, South Africa; Singh J., University of KwaZulu-Natal, Durban, South Africa; Stamm L., Merck, Rahway, NJ; Stranix-Chibanda L., University of Zimbabwe, Harare, Zimbabwe; Struble K., U. S. Food and Drug Administration, Silver Spring, MD; Townsend C. L., World Health Organization, Geneva, Switzerland; Van Wyk J., ViiV Healthcare, Washington, DC; Vannappagari V., ViiV Healthcare, Washington, DC; Wagner A., University of Washington, Seattle, WA; Warren M., AVAC, New York, NY; Zash R., Harvard University, Boston, MA.

## Glossary

ARVs: Antiretroviral therapies
PrEP: Pre-exposure Prophylaxis
FDA: U. S. Food and Drug Administration
IMPAACT: International Maternal Pediatric Adolescent AIDS Clinical Trials network
CAB-LA: Cabotegravir
TDF/FTC: Fumarate/Emtricitabine
DVR: Dapivirine
LEN: Lenacapavir
HIV: Human Immunodeficiency Syndrome
WHO: World Health Organization
PHASES: Pregnancy and HIV/AIDS: Seeking Equitable Study project
LMICs: Low-and-Middle-income countries
GPP: Good Participatory Practice
AMA: African Medicines Agency
BPCA: Best Pharmaceuticals for Children Act
PREA: Pediatric Research Equity Act
EU-M4ALL: European Union Medicine for all
EHRs: Electronic Health Records
FAERS: FDA Adverse Event Reporting System
PRGLAC: Taskforce on research specific to pregnant and lactating women
ASCoMP: WHO Advisory Committee on Safety of Medicinal Products
AU-3S: African Union-Smart Safety Surveillance
