# Supplementary material for: Advancing the inclusion of pregnant and lactating populations in HIV PrEP research: ethical, regulatory, and surveillance recommendations from a multisectoral working group
Source: Front Public Health. 2026 Jul 6;14:1811039. doi: 10.3389/fpubh.2026.1811039 (PMC13381502; doi:10.3389/fpubh.2026.1811039)
Supplement: Supplementary file 1 [file Data_Sheet_1.pdf]

Long-acting PrEP during pregnancy and lactation working group
Participant list

Abiodun Abiola
National Agency for Food and Drug Administration and Control, Abuja, Nigeria

Badshah Cyrus
Merck, Rahway, NJ

Belew Yodit
US Food and Drug Administration, Silver Spring, MD

Chan Hilda
Merck, Rahway, NJ

Chatani Manju
Independent Global Health consultant

Chi Benjamin
University of North Carolina at Chapel Hill, Chapel Hill, NC

Chigome Audrey
South African Health Products Regulatory Authority, Pretoria, South Africa

Davey Dvora
University of California, Los Angeles, CA

Day Suzanne
University of North Carolina at Chapel Hill, Chapel Hill, NC

Deaton Chris
Gilead Sciences, Inc. Foster City, CA

Delaney-Moretlwe Sinead
University of Witswatersrand, Johannesburg, South Africa

Donaldson Logan
Forum for Collaborative Research, Washington DC

Elemuwa Uchenna
National Agency for Food and Drug Administration and Control, Abuja, Nigeria

Escudeiro dos Santos Maria
European Medicines Agency Amsterdam

Force Lindsey
Gilead Sciences, Inc. Foster City, CA

Gonzalez Tome Maribel
European Medicines Agency, Madrid, Spain

Hamm Rush Sarah
Gates Foundation, Seattle, CA

Ikhide Yvonne
National Agency for Food and Drug Administration and Control, Abuja, Nigeria

Ingold Heather
World Health Organization, Geneva, Swtizerland

John-Stewart Grace
University of Washington, Seattle, WA

Kersey Kathryn
Gilead Sciences, Inc. Foster City, CA

Kumwenda Grace
AVAC, Southern Region, Malawi

Lamprianou Smaragda
World Health Organization, Geneva, Switzerland

Lataillade Max
Gates Foundation, Hartford, CT

Lievense Breanne
AVAC, New York

Love John
Merck, Rahway, NJ

Lyerly Anne
University of North Carolina at Chapel Hill, Chapel Hill, NC

Makura Cleopatra
Pangaea Zimbabwe, Harare, Zimbabwe

Matthews Lynn
University of Birmingham at Alabama, AL

Mgodi Nyaradzo
University of Zimbabwe, Harare, Zimbabwe

Miller Veronica
The Forum for Collaborative Research, Washington DC

Moodley Daya
University of Kwazulu-Natal, Durban, South Africa

Morroni Chelsea
University of Edinburgh, Edinburgh, Scotland

Mujugira Andrew
Makarere University, Kampala, Uganda

Mullick Charu
U.S. Food and Drug Administration, Silver Spring, MD

Ndagije Helen
National Drug Authority, Kampala, Uganda

Long-acting PrEP during pregnancy and lactation working group
Participant list

Ndembi Nicaise
International Vaccine Institute, Kigali, Rwanda

Noguchi Lisa
John Hopkins University, Baltimore, MD

Nwokike Jude
United States Pharmacopeia North Methesda, MD

Nyambayo Priscilla
Medicines Control Agency of Zimbawe, Harare, Zimbabwe

Osakwe Chukwunomso Ekene
The Forum for Collaborative Research, Washington DC

Plank Rebecca
Merck,Rahway, NJ

Prochazkz Nunez Mateo
World Health Organization, Geneva, Swtizerland

Ranade Roma
Forum for Collaborative Research, Washington DC

Renaud Francoise
World Health Organization, Geneva, Switzerland

Rinehart Alex
ViiV Healthcare, Washington DC

Robertson Michael
BDI Consulting, Nipomo, CA

Rodolph Michelle
World Health Organization, Geneva, Swtizerland

Friday Saidi
University of North Carolina Lilongwe, Lilongwe, Malawi

Schaefer Robin
Forum for Collaborative Research

Sehloho Tohlang
South African Health Products Regulatory Authority, Pretoria, South Africa

Semete Boitumelo
South African Health Products Regulatory Authority, Pretoria, South Africa

Singh Jerome
University of Kwazulu-Natal, Durban, South Africa

Stamm Luisa
Merck, Rahway, NJ

Stranix-Chibanda Lynda
University of Zimbabwe, Harare, Zimbabwe

Struble Kim
U.S. Food and Drug Administration, Silver Spring, MD

Townsend Claire Louise
World Health Organization, Geneva, Switzerland

Van Wyk Jean
ViiV Healthcare, Washington DC

Vannappagari Vani
ViiV Healthcare, Washington DC

Wagner Anjuli
University of Washington, Seattle, WA

Warren Mitchell
AVAC, New York

Zash Rebecca
Harvard University, Boston, MA
